# Apparent digestibility and calcium and phosphorus in urine after feeding different combinations of calcium and phosphorus sources to adult dogs

**DOI:** 10.1111/jpn.14038

**Published:** 2024-08-27

**Authors:** Celina Hofmann, Britta Dobenecker, Ellen Kienzle

**Affiliations:** ^1^ Department of Veterinary Science Chair of Animal Nutrition and Dietetics Munich Germany

**Keywords:** calcium, digestibility, dog, phosphorus, solubility

## Abstract

The present study aimed to investigate the effect of the combination of a water‐soluble calcium (Ca) source (CaCl_2_) with a water‐soluble phosphorus (P) source (NaH_2_PO_4_*2H_2_O, diet soluble, SOL) in comparison to a water‐insoluble P source (CaHPO4*2H_2_O, diet insoluble, INS) on apparent digestibility and renal excretion of Ca and P in dogs. The Ca intake was 226 mg/kg bodyweight (bw), the Ca/P ratio 1.9/1 in SOL and 2.0/1 in INS. The percentage of Ca from CaCl_2_ was 60% in SOL and 33% in INS. Eight adult Foxhound‐crossbred dogs FBI, (3–5 years, bw 24–32 kg) were available. Standard digestion trials were carried out (10 days adaptation, 5 days total faecal collection). Spontaneously excreted urine was collected pre‐ and postprandially. In vitro water solubility of P in the mineral premixes was determined. The Ca digestibility was negative in both trials without significant differences between the groups. Apparent P digestibility was increased in group SOL (26% vs. 20% in INS). In both groups, P content in urine was higher pre‐ compared to postprandial, with higher concentrations in group SOL. The same was true for the P/Crea ratio. The water solubility of P in the mineral premixes used in the trials showed considerable differences: The P in premix INS was insoluble in water after 1 and after 90 min. By contrast, the P in the premix SOL was highly soluble (98%) after 1 minute. After 90 min, however, the P solubility decreased to 43%, suggesting the formation of insoluble CaP salts, presumably from CaCl_2_ and NaH_2_PO_4_*2H_2_O. In conclusion, in the present study, apparent Ca digestibility in dogs was not affected by the solubility of Ca and P, while P digestibility and renal P excretion increased.

## 1 INTRODUCTION

Knowledge of dietary phosphorus (P) and calcium (Ca) availability is important for factorial calculation of requirements (Böswald et al., 2019) aiming for a sufficient supply without deficient or excessive intake. The latter is especially relevant to avoid adverse health effects caused by highly available P salts on renal, cardiovascular, and skeletal health in cats and dogs (Coltherd et al., 2019; Dobenecker et al., 2021a, 2021b; 2018).

The present study targeted the effects of either a highly water soluble or an insoluble P source in combination with partial exchange of common dietary Ca sources with soluble Ca chloride on the mineral balance. The hypothesis was that apparent digestibility of Ca and P as well as the renal excretion of P increases, when both minerals are given in a water‐soluble form.

## 2 ANIMALS, MATERIAL AND METHODS

For the study eight adult dogs (Foxhound‐crossbred, FBI, 3–5 years, 24–32 kg bodyweight (bw), five females, two neutered males, one intact male) were available. Digestion trials were carried out applying standard procedure: 3 days dietary change to test diet, 10 days adaptation, 5 days digestibility trial, individual feeding of one apportioned daily meal, individual housing for total faecal collection during digestibility trial, water ad libitum). In the first trial (insoluble, INS), the majority of Ca originated from CaHPO_4_*2H_2_O (60%) and CaCl_2_*2H_2_O (33%) with less than 1% from raw materials (Tables 1 and 2). CaHPO_4_*2H_2_O was used as inorganic P source because of its low solubility. After a > 2‐week wash‐out period with commercial dry maintenance dog food, a higher percentage of soluble Ca source (60% CaCl_2_, 39% CaCO_3_) was combined with a highly water‐soluble P source (NaH_2_PO_4_*2H_2_O) to create diet soluble (SOL).

**Table 1:** Diet composition and food intake (mean ± standard deviation) in group insoluble (INS) and soluble (SOL).

Diet	Unit	INS	SOL
Ingredients, basal diet	%	76.92 pork (rump with rind and fat, heart) 19.23 rice 3.85 gelatine	76.92 pork (rump with rind and fat, heart) 19.23 rice 3.85 gelatine
Dry matter	g/kg	391	391
Gross energy	MJ/kg dm	23.5	23.5
Crude protein	g/kg dm	398	398
Crude fat	g/kg dm	163	163
Crude fibre	g/kg dm	7	7
NfE	g/kg dm	416	416
Intake diet	g dm/kg bw	5.7 ± 0.6	5.7 ± 0.6
Intake lard	g dm/kg bw	2.3 ± 0.7	2.9 ± 0.9
Intake mineral supplement^a^	mg dm/kg bw	1259 ± 29	1718 ± 41
Vit D_3_, daily intake, total ration	IU/kg bw	14.5 ± 0.4	14.6 ± 0.4

Abbreviations: bw, bodyweight; dm, dry matter; NfE, nitrogen‐free extract; Vit D_3_, Vitamin D_3_.

^a^ Composition see Table 2.

**Table 2:** Composition of mineral premix (% dry matter) in group insoluble (INS) and soluble (SOL).

Additive	INS	SOL
CaHPO_4_*2H_2_O	42.064	0.000
CaCO_3_	0.000	14.289
CaCl_2_*2H_2_O	21.754	29.440
NaH_2_PO_4_*2H_2_O	0.000	29.498
NaCl	3.660	0.000
KCl	12.871	9.312
MgO	0.984	0.684
KJ	0.002	0.001
MnSO_4_*H_2_O	0.023	0.017
ZnSO_4_*7H_2_O	0.369	0.304
(CH_3_COO)_2_Cu*H_2_O	0.023	0.019
Na_2_SeO_3_*5H_2_O	0.002	0.001
Vit. A 1.000.000 IU/g	0.008	0.006
Vit. D_3_ 500.000 IU/g	0.002	0.002
Vit. E 50%	0.096	0.071
Vit. B_1_ 98%	0.004	0.003
Vit. B_2_ 80%	0.010	0.008
Vit. B_6_ 99%	0.002	0.002
Vit. B_12_ 0,1%	0.055	0.041
Niacin 100%	0.028	0.020
Choline 50%	5.366	3.993
Ca‐Pantothenic acid	0.025	0.019
Starch	12.652	12.368

To prevent weight loss, lard was given to those dogs with an energy requirement above the energy content of the basal diet. The mineral and vitamin premix were apportioned according to bw and added separately to the daily ration of each individual. This ensured identical mineral intake, with neglectable differences in mineral concentrations of the test diets for each dog. Both mineral supplements were tested for water‐soluble P by the method of Lineva et al. (2019) after the experiments because of unexpected results.

The dogs' faeces were lyophilised and ground after quantitative collection. After wet ashing, flame photometry was applied to determine Ca; P determination was carried out by photometry with ammonium molybdate and ammonium vanadate in HNO_3_ (GENESYS 10 UV, Thermo Spectronic, Rochester, NY, USA). Apparent digestibility (aD%) was calculated as follows: aD% = (Nutrient intake–faecal nutrient excretion)/nutrient intake *100. The Ca/P ratio of food and faeces was calculated. The difference between the Ca/P ratios in food and faeces (Δ) was calculated as follows: Ca/P in food–Ca/P in faeces.

Dietary cation‐anion‐balance (DCAB) was calculated using dm content of the minerals Ca, magnesium (Mg), sodium (Na), potassium (K), P, sulfur (S) and chloride (Cl) in the diet: DCAB (mmol/100 g dm) = 49.9*Ca + 82.3*Mg* + 43.5*Na + 25.6*K–64.6*P–62.4*S–28.2*Cl. The factor for S was calculated according to atomic weight and valence. (Behnsen, 1992; Kienzle et al., 1991; Kienzle & Wilms‐Eilers, 1994).

A quantitative collection of urine was not conducted due to ethical reasons. Spontaneous urine was caught ~2‐h preprandial and ~2‐hours postprandial while walking the dogs individually. Urine samples were pooled and analysed for total P and creatinine (Crea), with the latter one using the Jaffé method (MicroVue Creatinine Assay Kit, Quidel Corporation, reader: Sunrise Tecan).

The Shakiro‐Wilk normality test was used to assess the distribution of all means. Normally distributed means were compared by paired t‐test if applicable or Student's test. If data were not normally distributed, Welch t‐test (urine parameters) was used for comparison. Urine parameters which were sampled both pre‐ and postprandially in each group, were compared using a TWO WAY ANOVA with Mann–Whitney Rank Sum test as post hoc test. *p* < 0.05 was considered significant. The software SigmaPlot 12.5 (Systat Software GmbH) was used for statistical analysis.

The authors confirm that the ethical policies of the journal, as noted on the journal's author guidelines page, have been adhered to and the appropriate ethical review committee approval has been received. The authors confirm that they have followed EU standards for the protection of animals used for scientific purposes. All procedures and protocols were conducted in accordance with the European guidelines of the Protection of Animals Act. The study was approved by the responsible animal protection officer of the Faculty of Veterinary Medicine of the Ludwig‐Maximilians‐Universität München, as well as the Government of Upper Bavaria (reference number 55.2‐1‐54‐2532‐202‐2016).

## 3 RESULTS

**Figure 1:**
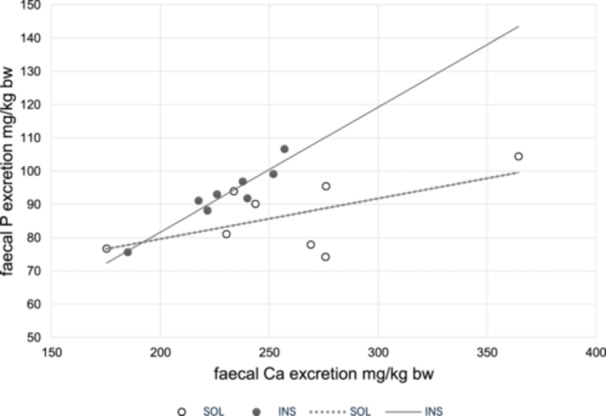
Regression between faecal Ca‐excretion and faecal P‐excretion in group (INS ● ‒; *y* = 0.3755× + 6.5598, *R* ^2^ = 0.9036) and group (SOL ○ ┉; *y* = 0.1211× + 55.423, *R* ^2^ = 0.3676).

Throughout the study, all animals remained healthy. Dry matter (dm) intake, apparent dm digestibility, and energy digestibility are given in Table 3. The apparent Ca digestibility was negative in both trials (Table 3) without statistical differences. The same applies to the apparently digested Ca. There was a negligible difference in the P intake (1.7%) between both trials (Table 3). The apparent P digestibility was overall moderate, but significantly higher in diet SOL (*p* = 0.011); the same was true for the apparently digested P (*p* = 0.008). This resulted in a significant difference in the faecal Ca/P ratio. The same was true for delta (Table 3). The regression between faecal Ca and P excretion is given in Figure 1. The relationship was very close in group INS and practically lost in group SOL.

**Table 3:** Intake, apparent digestibility, and faecal excretion of dry matter, calcium and phosphorus, dietary cation‐anion‐balance (mean ± standard deviation) in group insoluble (INS) and soluble (SOL).

Diet	Unit	INS	SOL	*p*‐values
Total dm intake	g dm/kg bw	9.2 ± 0.8	10.3 ± 1.0	0.0012**
Faecal dm excretion	g/kg bw	1.4 ± 0.2	1.7 ± 0.4	0.023*
Apparent dm digestibility	%	85 ± 5	83 ± 3	
Apparent energy digestibility	%	93 ± 2	92 ± 2	0.028*
Ca intake	mg/kg bw	226 ± 5	226 ± 6	
Soluble Ca	% of Ca intake	33	60	
Faecal Ca excretion	mg/kg bw	240 ± 50	259 ± 51	
Apparently digested Ca	mg/kg bw	−4 ± 18	−33 ± 47	
Apparent Ca digestibility	%	−2 ± 8	−14 ± 20	
P intake	mg/kg bw	115 ± 4	117 ± 4	<0.0001***
Soluble P	% of P intake	0	84	
Faecal P excretion	mg/kg bw	93 ± 8	87 ± 10	0.0028*
Apparently digested P	mg/kg bw	22 ± 6	30 ± 8	0.008**
Apparent P digestibility	%	20 ± 6	26 ± 7	0.011*
Ca/P total diet		2.0	1.9	
Ca/P faeces		2.5 ± 0.1	3.0 ± 0.5	0.023*
Δ Ca/P total diet – Ca/P faeces		−0.5 ± 0.1	−1.1 ± 0.5	0.018*
Dietary cation‐anion‐balance	mmol/kg dm	−80 ± 21	−53 ± 18	

Abbreviations: bw, bodyweight; Ca, calcium; dm, dry matter; P, phosphorus.

* Significant (*p* < 0.05).

** Highly significant (*p* < 0.01).

*** Very highly significant (*p* < 0.001).

In both diets, the P content in the urine was higher before than after feeding (Table 4). It was higher in group SOL throughout, leading to a significant interaction (Table 4). The urinary Crea content depended on time of sampling and diet (Table 4), with a significant interaction. In principle, it was higher in preprandial samples; the same was true for the P/Crea ratio (Table 4). Urinary Ca content was not affected by the diet (Table 4). The Ca/Crea ratio as well as the urinary pH was lower before than after feeding in both trials (Table 4). Diet SOL with higher amounts of CaCl_2_ reduced the urinary pH both pre‐ and postprandial.

**Table 4:** Urinary pH and concentration of creatinine, calcium, and phosphorus (mean ± standard deviation) in group insoluble (INS) and soluble (SOL).

Parameter		INS		SOL	
Time of sampling	Unit	Preprandial	Postprandial	Preprandial	Postprandial
pH^a^		5.8 ± 0.2	5.4 ± 0.3	5.2 ± 0.2	4.9 ± 0.3
		*n* = 25	*n* = 27	*n* = 28	*n* = 31
Crea^a^^,^ ^c^	mmol/L	11.5 ± 3.0	8.0 ± 3.1	13.3 ± 5.3	7.1 ± 2.5
		*n* = 25	*n* = 28 (9)	*n* = 29 (1)	*n* = 33 (10)
Ca	mmol/L	2.6 ± 1.6	3.0 ± 1.5	2.8 ± 1.4	2.9 ± 1.2
		*n* = 25 (4)	*n* = 27 (5)	*n* = 27	*n* = 33 (1)
P^a^^,^ b ^,^ c	mmol/L	31.7 ± 14.5	5.5 ± 2.0	47.7 ± 27.3	7.0 ± 4.7
		*n* = 23	*n* = 28 (23)	*n* = 29	*n* = 34 (22)
Ca/Crea^a^		0.2 ± 0.1	0.4 ± 0.2	0.2 ± 0.1	0.4 ± 0.2
		*n* = 25	*n* = 27	*n* = 27	*n* = 33
P/Crea^a^ ^,^ b		2.7 ± 1.0	0.8 ± 0.3	3.7 ± 1.4	0.1 ± 0.8
		*n* = 23	*n* = 28	*n* = 29	*n* = 33

Abbreviations: Ca, calcium; crea, creatinine; *n*, number of samples P, phosphorus; (number of values below detection limit in brackets).

^a^ Significant difference between times of sampling within group.

^b^ Significant difference between groups.

^c^ Significant interaction.

The in vitro water solubility of the mineral supplements used in the trials showed considerable differences: INS was insoluble in water after 1 and after 90 min while SOL showed a high percentage of P soluble after 1 min (98%). After 90 min, however, the P solubility decreased to 43%.

## 4 DISCUSSION

In the present study, the apparent Ca digestibility of diet SOL containing soluble Ca and P sources did not differ significantly from diet INS with mostly insoluble Ca and P sources. This confirms previous results on uniformity of Ca digestibility in dogs, which appears to be rather independent of the Ca source (Böswald et al., 2018; Mack et al., 2015).

The study of Schünemann et al. (1989) suggests that in dogs the main site of Ca absorption is the large intestine. It is quite likely that by the time the Ca reaches the large intestine, it is no longer water‐soluble CaCl_2_. There may be a falling‐out of low water‐soluble Ca salts such as CaCO_3_, CaHPO_4_. This would explain that there is no difference between Ca sources.

Ca content as well as Ca/Crea in the urine was unaffected by diet. There was, however, an effect of time after feeding on the Ca/Crea ratio as well as the urinary pH. It is known that during metabolic acidosis the renal Ca excretion increases (Alexander et al., 2016). In the present study, the postprandial urinary pH was always lower after feeding SOL, thus reflecting the intake of acidifying salts such as CaCl_2_, which also confirms previous studies in dogs (Behnsen, 1992).

**Figure 2:**
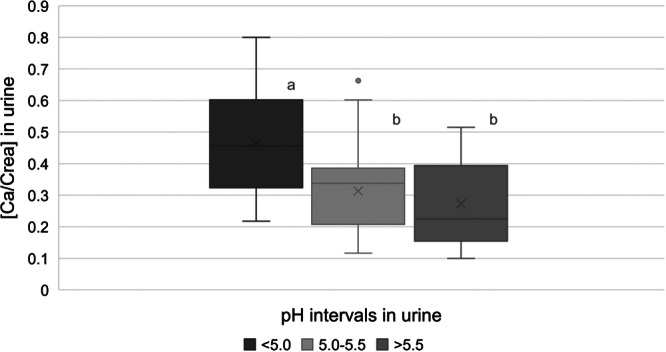
Boxplots (median, 25/75% and 5/95%, outliers) of the ratio of Ca/Crea in urine (mmol/L) in relation to urinary pH. Medians not sharing a superscript letter are significantly different.

There was a clear‐cut effect of urinary pH on the urinary Ca/Crea coefficient, which was independent of the feeding. Especially when the pH was below 5, the Ca/Crea coefficient was significantly increased (Figure 2). According to Alexander et al. (2016), the increase of renal Ca excretion during acidosis is due to Ca mobilisation from the skeleton. This would explain why there was no specific effect of the diet apart from the effect of urinary pH. It also agrees with the negative Ca balance in both trials.

On the contrary to the Ca excretion, P excretion was affected significantly by diet composition. Apparent P digestibility increased significantly when feeding more soluble Ca and P sources (SOL). The same was true for the preprandial urinary P concentration as well as the P/Crea (Table 4), which suggests an increase in fractional as well as absolute renal P excretion in group SOL. This increases the P load per nephron, which is an important determinant of P nephrotoxicity (Haut et al., 1980).

Compared to previous studies on canine P excretion (Dobenecker et al., 2021a, 2021b; Herbst, 2021), the statistically significant increase in apparent digestibility and renal P excretion was relatively small. Given the statistical significance of the difference in P digestibility the smaller increase of digestibility compared to previous studies is unlikely to be related to the number of animals in the trials. A number of 8 is more than enough to find differences in apparent digestibility of major minerals. This has been shown in previous studies (Dobenecker et al., 2021b).

The decrease of solubility of P from the premix SOL after 90 min in water, might help to explain the difference to previous studies. If both minerals are given in a highly soluble form, it is possible that they form more or less water‐insoluble complexes such as CaHPO_4_ which are then precipitated and less available. The same thing might happen during the passage of the food through the gastrointestinal tract. There may be other factors such as breed differences as well. Kiefer‐Hecker et al. (2018) demonstrated a difference of the Ca and P excretion between growing beagles and FBI dogs. Even so in the present study in the diet containing soluble inorganic P, faecal P excretion in relation to faecal Ca excretion was decreased. Given the close connection between these two minerals this is likely to have metabolic consequences.

In summary, Ca excretion in dogs is rather unaffected by Ca source, whereas P excretion can be influenced considerably by P source and/or Ca source.

## CONFLICT OF INTEREST STATEMENT

The authors declare no conflicts of interest.

## Data Availability

Data supporting this study are not publicly available. Please contact Celina.Hofmann@gmx.de.
